# UNet based on dynamic convolution decomposition and triplet attention

**DOI:** 10.1038/s41598-023-50989-2

**Published:** 2024-01-02

**Authors:** Yang Li, Bobo Yan, Jianxin Hou, Bingyang Bai, Xiaoyu Huang, Canfei Xu, Limei Fang

**Affiliations:** 1 Academy for Advanced Interdisciplinary Studies, Northeast Normal University, Changchun, 130024 Jilin China; 2 Shanghai Zhangjiang Institute of Mathematics, Shanghai, 201203 China; 3 School of Computer Science and Engineering, Changchun University of Technology, Changchun, 130012 Jilin China; 4 Pazhou Lab, Guangzhou, China; 5 The Third Hospital of Jilin University, Changchun, 130117 Jilin China; 6 Encephalopathy Center, The Third Affiliated Hospital of Changchun University of Chinese Medicine, Changchun, 130117 Jilin China

**Keywords:** Network models, Image processing

## Abstract

The robustness and generalization of medical image segmentation models are being challenged by the differences between different disease types, different image types, and different cases.Deep learning based semantic segmentation methods have been providing state-of-the-art performance in the last few years. One deep learning technique, U-Net, has become the most popular architecture in the medical imaging segmentation. Despite outstanding overall performance in segmenting medical images, it still has the problems of limited feature expression ability and inaccurate segmentation. To this end, we propose a DTA-UNet based on Dynamic Convolution Decomposition (DCD) and Triple Attention (TA). Firstly, the model with Attention U-Net as the baseline network uses DCD to replace all the conventional convolution in the encoding-decoding process to enhance its feature extraction capability. Secondly, we combine TA with Attention Gate (AG) to be used for skip connection in order to highlight lesion regions by removing redundant information in both spatial and channel dimensions. The proposed model are tested on the two public datasets and actual clinical dataset such as the public COVID-SemiSeg dataset, the ISIC 2018 dataset, and the cooperative hospital stroke segmentation dataset. Ablation experiments on the clinical stroke segmentation dataset show the effectiveness of DCD and TA with only a 0.7628 M increase in the number of parameters compared to the baseline model. The proposed DTA-UNet is further evaluated on the three datasets of different types of images to verify its universality. Extensive experimental results show superior performance on different segmentation metrics compared to eight state-of-art methods.The GitHub URL of our code is https://github.com/shuaihou1234/DTA-UNet.

## Introduction

Magnetic resonance imaging (MRI) and computed tomography (CT) have become the main ways for clinicians to analyze disease. The purpose of medical image segmentation is to segment lesion regions or lesion organs from different types of medical images, and provide an important basis for accurate assessment, diagnosis and treatment of subsequent diseases. For example, the segmentation of ischemic stroke on MRI images can help clinicians quickly locate and quantify the lesion, so as to determine the ischemic penumbra as soon as possible, in time for timely clinical rescue and reduce medical expenses. It can also reduce patient injuries and improve the quality of life and survival rate of patients. Segmentation of lesions in CT images of Coronavirus Disease 2019 (COVID-19) can help clinicians locate and quantify lesions, prevent missed and false detection, and assist clinicians in timely diagnosis of patients.

At present, the classic segmentation models include U-Net^1^, DeepLab^2^, PSPNet^3^ etc., which have achieved good results in the lesion segmentation of medical images^4–6^. U-Net^1^ adopted an encoder-decoder structure, the encoder is used to extract feature information, and the decoder is used to restore feature information. In addition, the high-resolution information and deep-level semantic information are fused by skip connection, which enriches the context information and achieves success in small-scale dataset segmentation. However, U-Net typically exhibits limitations in explicitly modeling long-range dependencies and may lead to a decrease in resolution and loss of information due to the use of convolution and pooling operations. DeepLab^2^ first proposed atrous convolution, which expanded the receptive field without adding additional params. In addition, it also used a fully connected Conditional Random field to enhance the model’s ability to locate the boundary. This method has developed rapidly in the fields of natural image segmentation and medical image segmentation. However, DeepLab has poor semantic understanding ability for objects of different sizes and is not suitable for segmenting lesions with large size variations. PSPNet^3^ proposed a pyramid pooling module based on the encoder-decoder structure, which can realize multi-scale information fusion and help the model obtain richer global context information. Recently, some literatures^7,8^ have used Transformer to model long-range dependencies, but they require large amounts of training data and significant computational resources due to the large number of query operations, which limits their practical application in clinical settings. Zhu et al.^9^ proposed a lightweight 3D segmentation network, SV-net, which replaces the convolutional blocks in V-Net with lightweight convolutional blocks, significantly speeding up the training process. To reduce the dependence of deep learning networks on labels, Liu et al.^10^ proposed a weakly supervised biomarker localization and segmentation network that only requires image-level annotations. This network uses a novel pretraining strategy based on supervised contrastive learning, effectively reducing the annotation burden on ophthalmologists in OCT images.

In lesion segmentation, the amount of medical data containing real labels is small, so among the above networks, U-Net, which is friendly to small-scale dataset segmentation, is the most widely used and shows great potential. Zhou et al.^4^ redesigned the skip connection to make up for the semantic difference between the encoder-decoder feature maps. The authors also used model pruning to reduce the complexity of the model and achieve a balance between inference time and performance. Gu et al.^11^ added a context extractor module on the basis of the encoder-decoder structure to solve the problem of spatial information loss caused by continuous convolution operations and pooling operations in the original U-Net. Oktay et al.^12^ proposed the Attention U-Net, embedding the AG module into the U-Net skip-connections for the first time. The AG module can effectively suppress feature activation in irrelevant regions, so as to improve the segmentation accuracy of the model. Cai et al.^13^ used Attention U-Net as the baseline network, and connected the features fused at different scales with the parallel spatial attention (SA) mechanism and channel attention (CA) mechanism, effectively capturing the long-range dependence relationship. Mu et al.^14^ proposed an attention-based residual U-Net framework for intracranial aneurysm segmentation. This framework introduces differential preprocessing and geometric post-processing functions, which can automatically segment intracranial aneurysms with high accuracy. U-Net and its variants have achieved success in different medical image segmentation tasks. However, the conventional convolution operation and simple skip connections in U-Net limit the performance of the model. It is not conducive to deal with the problem of lesions with large differences in shape, size or location, and is sensitive to the problem of blurred boundaries. This is a common problem in lesion segmentation of different types of diseases presented in different images, and it is extremely challenging to build a universal model with multi-type lesion segmentation. To address the above problems, we propose a DTA-UNet that can effectively segment different types of lesions and is more friendly to the boundary blurring problem. The main contributions are as follows:

1. DCD was introduced to replace all the regular convolutions in the encoding-decoding process of Attention U-Net^12^, which significantly improving the feature extraction capability of the CNN with a small increase in parameters.

2. We use the combination of TA and AG to act on the skip connection, highlight the lesion region, and optimize the features extracted by dynamic convolution.

3. A U-Net Based on DCD and TA is called DTA-UNet, which is a universal segmentation model that is friendly to lesion regions as well as boundaries, and shows accurate segmentation results on three types of datasets.

The rest of the paper is organized as follows: “Related work” provides a brief background on dynamic convolution methods and attention mechanisms. “Universal lesion segmentation framework DTA-UNet” provides a detailed overview of the proposed DTA-UNet methodology, and discusses the architecture diagram of the proposed DTA-UNet methodology as well as the TA structure diagram. In “Experiments”, detailed information about the dataset is provided. The proposed DTA-UNet method is compared with the use of various state-of-art deep learning models, and the experimental results are discussed. Finally, “Conclusion” provides a number of conclusions and recommendations for future work.

## Related work

### Dynamic convolution

Before the advent of dynamic convolution, conventional convolution used the same convolutional kernel parameters for all input images, which limited the feature expression ability of the network. People usually enhance the expressive ability of the network by increasing the depth or width of the network. However, this method will greatly increase the calculation cost. Therefore, Yang et al.^15^ proposed a Conditionally parameterized Convolutions (CondConv), which applies the attention mechanism to several parallel convolution kernels, breaking the characteristics of conventional convolution for all input shared params. It shows excellent performance on detection and classification tasks, and provides a new direction for improving the expressiveness of the model. Chen et al.^16^ proposed DYnamic Convolution (DY-Conv) on the basis of CondConv. By constraining attention and introducing the temperature parameter into Softmax, the training speed of the model was accelerated and the number of params was reduced. Li et al.^17^ analyzed the problems of the large number of dynamic convolution params and the high difficulty of joint optimization of dynamic attention and conventional convolution kernels from the perspective of matrix decomposition, proposed a DCD model with dynamic channel fusion mechanism. Compared with CondConv and DY-Conv, DCD has smaller params and better performance. Therefore, in this study, all the conventional convolution of the encoder-decoder part of Attention U-Net was replaced by DCD, with a small number of params in exchange for a large performance improvement.

### Attention mechanism

In the field of computer vision, the attention mechanism is to calculate different weight params for the input feature map, so that the model pays more attention to key information and ignores irrelevant information such as background. Hu et al.^18^ proposed the Squeeze-and-Excitation (SE) block to realize information interaction in the channel dimension. However, the calculation of the excitation part is mainly realized through the operation of dimension reduction and dimension increase, and the operation of dimension reduction is not conducive to the effective learning of channel relations by the network. Wang et al.^19^ proposed Efficient CA (ECA), which removes the dimensionality reduction operation and realizes the information interaction between some channels through one-dimensional convolution, reducing the number of params and improving performance. Yang et al.^20^ proposed a Simple parameter-free Attention Module (SimAM). The core is to use the energy function to mine the importance of neurons. The lower the energy, the higher the importance. The method shows good performance on both detection and segmentation tasks. Liu et al.^21^ proposed the Normalization-based Attention Module (NAM), which represents the importance of channels and spaces through the variance in Batch Normalization (BN). The larger the variance, the richer the channel or spatial information, and the higher the importance. Woo et al.^22^ proposed the Convolutional Block Attention Module (CBAM), which combines the SA mechanism and the CA mechanism. CBAM can adaptively optimize features and seamlessly connect to the CNN architecture. However, SA and CA in CBAM are independent of each other, and lack cross-dimension information that is beneficial to performance. Therefore, Misra et al.^23^ proposed a near parameter-free TA, which uses the residual connection and the rotation transformation between tensors to realize the information interaction across the three dimensions of channel, height and width. At the same time, it avoids the adverse effects caused by the dimensionality reduction operation in the CBAM module. Compared with the above attention mechanisms, TA not only realizes the attention of spatial and channel dimensions, but also realizes the cross-dimension information interaction. Therefore, we selected TA to optimize the features. In addition, in medical image segmentation, since the medical image background accounts for a large proportion, and the great difference in the shape and size of the segmentation target itself. In this regard, this study designed a combination of TA and AG, which not only focused on lesions of different shapes and sizes, but also eliminated the adverse effects caused by irrelevant regions and backgrounds in skip connections.

## Universal lesion segmentation framework DTA-UNet

**Figure 1:**
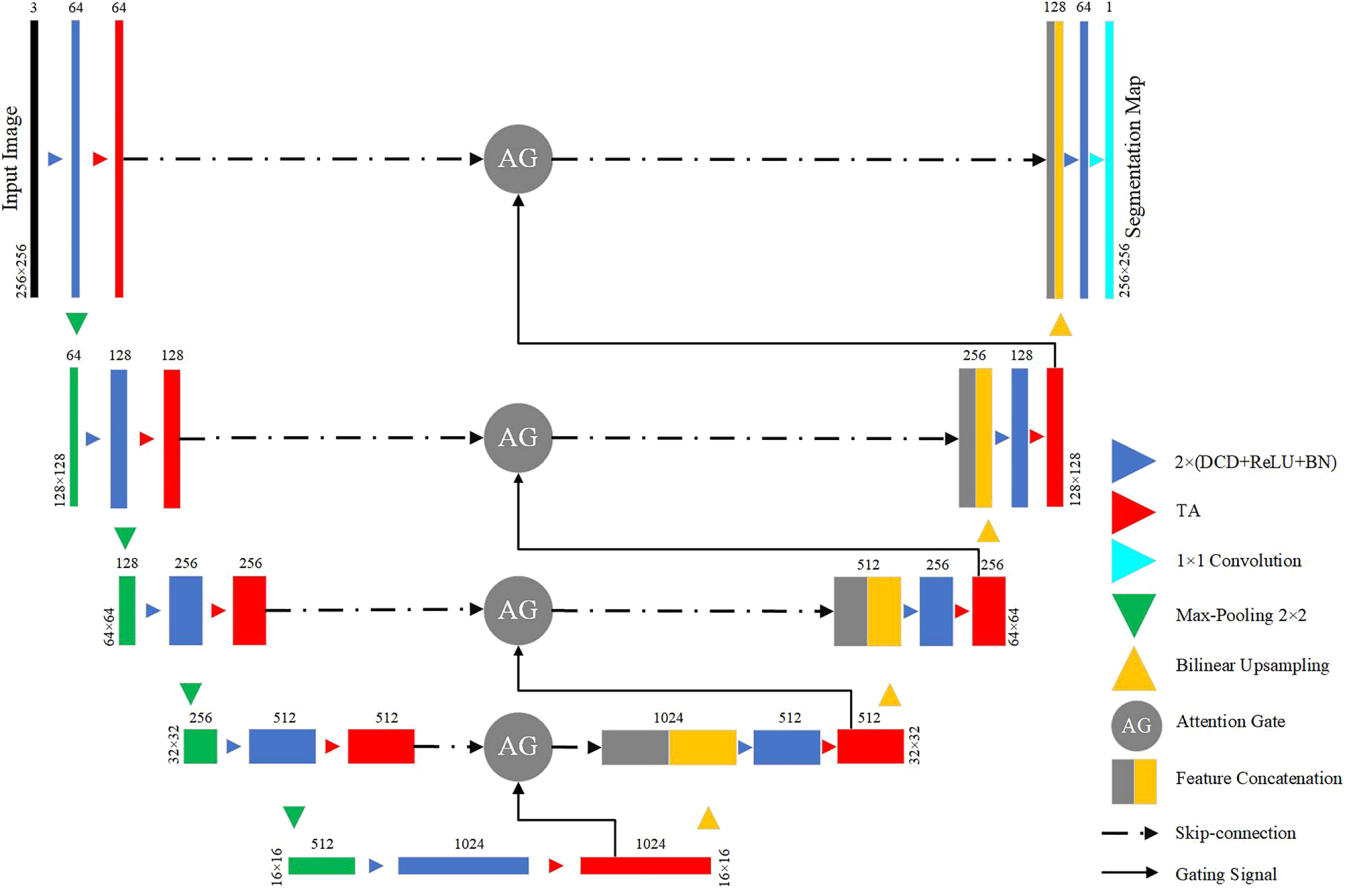
The architecture of DTA-UNet. Each rectangular box represents a feature map, and the number directly above the rectangular box represents the number of channels of the feature map. In the encoding stage, the number in the lower left part of the rectangular box represents the size of the feature map. In the decoding stage, the number in the lower right part of the rectangular box represents the size of the feature map.

The proposed universal lesion segmentation framework DTA-UNet is shown in Fig. 1, with the Attention U-Net containing the AG structure as the basic network. First, in order to enhance the feature expression ability of the model, we use DCD to replace all the conventional convolutions in the encoding-decoding process. Secondly, we use the combination of TA and AG to correct the feature maps extracted by DCD, and eliminate the ambiguity caused by irrelevant regions in spatial and channel dimensions. Finally, the output is obtained by bilinear upsampling.

DTA-UNet adopts a conventional encoder-decoder structure. The encoder consists of dual DCD, TA and max pooling, which are used to extract features, optimize features, reduce image resolution and increase the number of channels. The decoder consists of bilinear upsampling, dual DCD, skip connection, TA and $1\times 1$ convolution, which is used to restore the encoded abstract feature map to its original size.

The conventional CNN is limited by the computation cost, and its network depth and width can not be greatly increased, which leads to its limited expressive ability. We replace the conventional convolutions in the encoding-decoding process with DCD, which helps the network to obtain finer semantic information and effectively balances the computational cost and expression ability. DCD replaces dynamic attention with dynamic channel fusion. The basic principle of DCD is as follows, and the dynamic convolution proposed by CondConv and DY-Conv is shown in Eq. (1).

1 $$\begin{aligned} {\textrm{W}({\textbf{x}}) = \sum \limits _{k = 1}^K {{\pi _k}} ({\textbf{x}}){{\textrm{W}} _k}} \end{aligned}$$

Where $\textbf{x}$ represents the input feature graph, ${\textrm{W}}({\textbf{x}})$ represents the dynamic convolution kernel, ${{\textrm{W}} _k}$ represents the k-th conventional convolution kernel, *k* represents the number of convolution kernels, and the value range is $\mathrm{{[}}1,K\mathrm{{]}}$, ${\pi _k}{\textbf{x}}$ the attention weight coefficient, and the value range is $\mathrm{{[}}0,1\mathrm{{]}}$, in addition, constraints $\sum _{k = 1}^K {{\pi _k}} ({\textbf{x}}\mathrm{{) = 1}}$.

First, by representing each conventional convolution with the idea of residuals, we can get Eq. (2):

2 $$\begin{aligned} {{{\textrm{W}} _k} = {{\textrm{W}} _0} + \Delta {{\textrm{W}} _k}} \end{aligned}$$

Where the definition of ${{\textrm{W}} _k}$ is the same as that defined in Eq. (1), denotes the k-th regular convolution kernel, ${{\textrm{W}} _0} = \sum _{k = 1}^K {{{\textrm{W}} _k}}$ denotes the mean convolution kernel.

Secondly, the singular value decomposition of $\Delta {{\textrm{W}} _k}$ is performed as shown in Eq. (3):

3 $$\begin{aligned} {\Delta {{\textrm{W}} _k} = {{\textrm{W}} _k} - {{\textrm{W}} _0} = {{\textrm{U}} _k}{{\textrm{S}} _k}{{\textrm{V}} _k}^T} \end{aligned}$$

Where $\Delta {{\textrm{W}} _k}$,${{\textrm{W}} _k}$,${{\textrm{W}} _0}$ and have the same meaning as above, ${{\textrm{U}} _k}$ represents the left singular matrix corresponding to $\Delta {{\textrm{W}} _k}$, ${{\textrm{S}} _k}$ representing the diagonal matrix, and ${{\textrm{V}} _k}$represents the right singular matrix.

Then, by bringing Eq. (3) into Eq. (2), we get the entire dynamic convolution, as shown in Eq. (4):

4 $$\begin{aligned} {{\textrm{W}}({\textbf{x}}) = \sum \limits _{k = 1}^K {{\pi _k}} ({\textbf{x}}){{\textrm{W}} _0} + \sum \limits _{k = 1}^K {{\pi _k}} ({\textbf{x}}){{\textrm{U}} _k}{{\textrm{S}} _k}{{\textrm{V}} _k}^T = {{\textrm{W}} _0} + {\textrm{U}}{} \mathbf{\pi }({\textbf{x}}){{\textrm{SV}} ^T}} \end{aligned}$$

Where ${{\textrm{W}} _0}$ is expressed the same as above, ${\textrm{U}}$ is the left singular matrix of ${\textrm{W}}({\textbf{x}})$, the shape is ${\textrm{U}} = \mathrm{{[}}{{\textrm{U}} _1},...,{{\textrm{U}} _K}\mathrm{{]}}$, ${\textrm{S}}$ is the unit diagonal matrix, which represents the convolution weight coefficient obtained by the attention mechanism, and the shape is ${\textrm{S}} = \mathrm{{[}}{{\textrm{S}} _1},...,{{\textrm{S}} _K}\mathrm{{]}}$, ${\textrm{V}}$ is the right singular matrix of ${\textrm{W}}({\textbf{x}})$, and the shape is ${\textrm{V}} = \mathrm{{[}}{{\textrm{V}} _1},...,{{\textrm{V}} _K}\mathrm{{]}}$.

Thirdly, if the constraint $\sum _{k = 1}^K {{\pi _k}} ({\textbf{x}}\mathrm{{) = 1}}$ is relaxed and the channel dimension is used to represent the dynamic convolution, the number of channels of a single regular convolution is ${\textrm{c}}$, then the number of channels of ${\textrm{K}}$ regular convolutions is ${\textrm{KC}}$, as shown in Eq. (5):

5 $$\begin{aligned} {{\textrm{W}}({\textbf{x}}) = \Lambda ({\textbf{x}}){{\textrm{W}} _\mathrm{{0}}} + \sum \limits _{i = 1}^{KC} {{\pi _{\Big \lceil {{i/ C}} \Big \rceil }}} ({\textbf{x}}){{\textrm{u}} _i}{{\textrm{s}} _i}{_\mathrm{{,}}{}_i}{{\textrm{v}} _i}^{\textrm{T}}} \end{aligned}$$

Where ${{\textrm{u}} _i},{{\textrm{v}} _i}$ are vectors, and ${{\textrm{s}} _i}{_\mathrm{{,}}{}_i}$ is the diagonal matrix.

Finally, the dimension of the hidden space is artificially set to ${\textrm{L}}$, and ${\mathrm{L<<C}}$. What’s more ${\textrm{P}}$, $\Phi ({\textbf{x}})$, and ${{\textrm{R}} ^T}$ are used to replace Eq. (5). The expression of the whole DCD is shown in Eq. (6):

6 $$\begin{aligned} { W (x)=\Lambda (x) W_0 + P \Phi (x) R^T } \end{aligned}$$

Where *x* represents the input feature map, *W*(*x*) and $W_{0}$ are both $C\times k^2$ order matrix, C represents the number of channels, and $k^2$ represents the convolution kernel size is $k\times k$. W(x) represents DCD, $\Lambda (x)$ is a $C\times C$ diagonal matrix, generated by attention, and $W_{0}$ is the mean convolution kernel. R is the $k^2\times L$ order matrix, the purpose is to compress the number of kernel elements from $k^2$ to $L$. $\Phi (x)$ is a $L\times L$ matrix, and its function is to dynamically fuse $L$ elements , which can significantly reduce the dimension of the hidden space. $P$ is the $C\times L$ order matrix, used to increase the dimension. Artificially setting $L =\lfloor k^2/2 \rfloor$ to reduce the number of params. In this paper, $k =3$, $L =4$.

**Figure 2:**
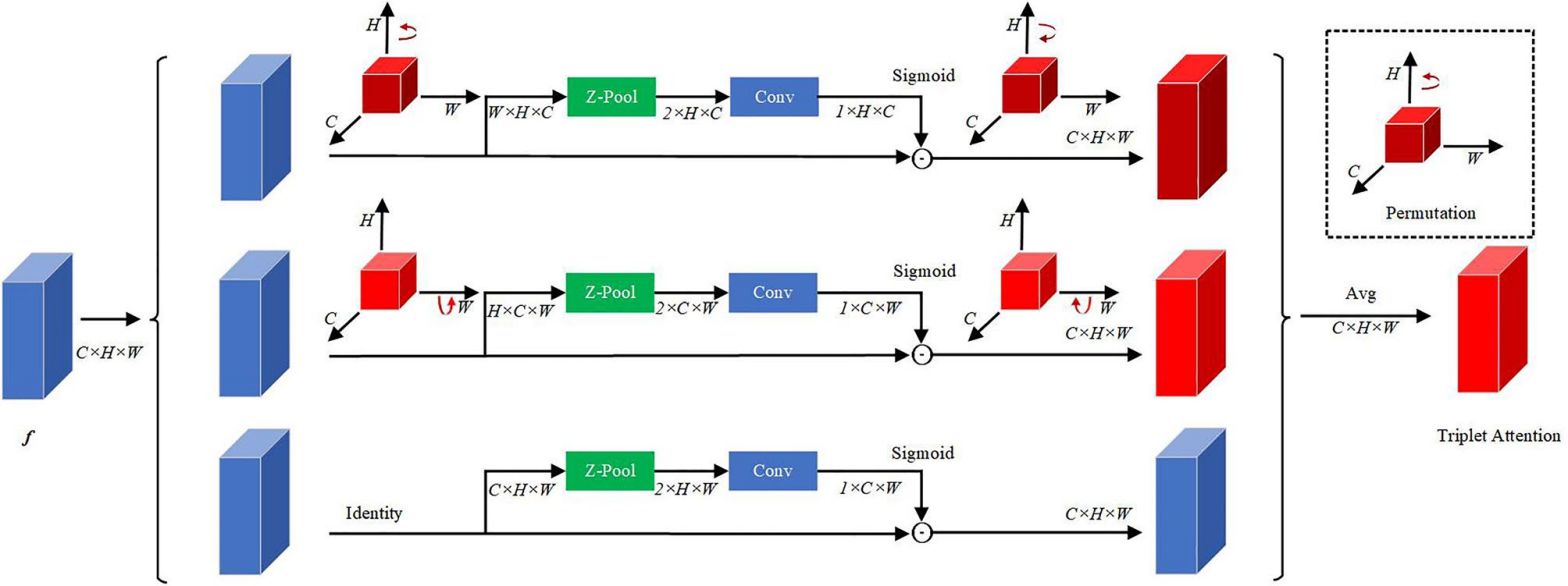
The architecture of TA.

Furthermore, in order to further optimize the features, we add TA in the encoding-decoding process to make it more focused on segmentation targets. Assume the feature extracted by DCD that is *f*. It can be expressed as $f=x \odot W(x)$, $\odot$ represents element-wise multiplication. The shape of *f* is $C\times H\times W$, where C is the number of channels, H is the height, and W is the width, the structure of the TA is shown in Fig. 2.

It is a three-branch structure. The first and second branches are relatively similar, mainly through rotation, Z-Pool, convolution and Sigmoid to realize the dimensional interaction with between *C*,*H* and *C*,*W*. The third branch is used to calculate spatial attention weights. Finally, the three branches are averaged. The feature map obtained in this way not only has the advantages of CA and SA, but also has cross-dimensional interactive information, which realizes the purpose of optimizing features. The Z-pool^23^ layer is responsible for reducing the tensor of the C dimension to 2 dimensions, connecting the average and maximum pooling features on this dimension. This allows the layer to retain a rich representation of the actual tensor, while reducing its depth to make further computationally less intensive. The expression of Z-Pool is shown in Eq. (7):

7 $$\begin{aligned} Z-Pool(f)=[Maxpool_{0d}(f),Avgpool_{0d}(f)] \end{aligned}$$

where Maxpool$_{0d}(f)$, Avgpool$_{0d}(f)$ represents that max pooling and average pooling are performed on the 0 dimension.

Algorithm 1 shows the pseudocode of the model training process proposed in this paper.

**Algorithm 1:**
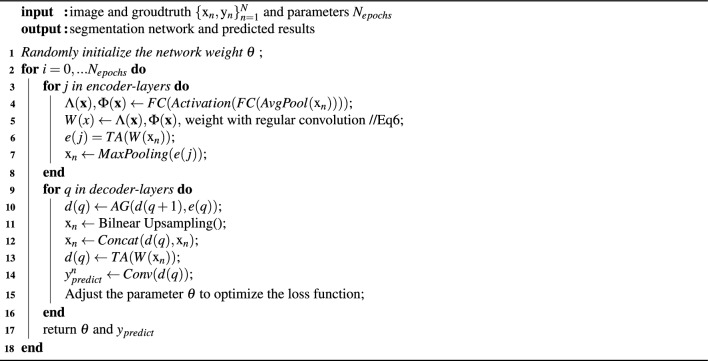
Train DTA-UNet.

## Experiments

### Datasets and evaluation metrics

#### Dataset description

In order to evaluate the generalization ability and robustness of the DTA-UNet, this paper will conduct experiments on three different types of datasets. The three datasets have the following things in common: (1) The size, shape and location of the lesion are quite different, (2) The boundary of the lesion is blurred. The three datasets are described in detail as follows:

**AIS Dataset** This dataset is the MRI image of stroke patients provided by the cooperative hospital, referred to as the AIS dataset. This dataset contains MRI images of 132 ischemic stroke patients. About 20 axial slices with a resolution of $160\times 160$ were obtained for each patient, for a total of 2644 axial slices. The labels were annotated by neurosurgeons from a cooperative hospital using ITK-SNAP 3.8.0 software. In this paper, 771 images containing lesions were selected from 2644 images, which were unified into $256\times 256$, and the training set and testing set were divided according to the ratio of 8:2.

**COVID-SemiSeg Dataset** This dataset is a public dataset for the COVID-19 segmentation provided by Fan et al.^24^, which contains 1698 two-dimensional CT images with different resolutions. Among them, 98 CT images are ground truth (GT) marked by doctors, and 1600 CT images belong to pseudo labels generated by semi-supervision. The dataset is linked as: https://drive.google.com/file/d/1bbKAqUuk7Y1q3xsDSwP07oOXN_GL3SQM/view. In this paper, the unified size of 1698 two-dimensional CT images with different resolutions is $256\times 256$. We use 50 two-dimensional CT images of COVID-19 with GT and 1600 two-dimensional CT images of COVID-19 with pseudo-labels as the training set, and 48 images with GT as the testing set.

**ISIC 2018 Dataset**^25^ This dataset is a public dataset released by the International Skin Imaging Collaboration (ISIC) in 2018. A total of 2598 labeled two-dimensional dermoscopic images. Resolutions vary in size, such as $767\times 576$, $6688\times 4459$ etc. The dataset is linked as: https://challenge.isic-archive.com/data/#2018. In this paper, We unify the size of all images to $256\times 256$, and divide the training set and testing set according to the ratio of 8:2.

#### Evaluation indicators

We selected Intersection over Union (IoU), Dice Similarity Coefficient (DSC), Hausdorff Distance (HD) and Precision (Pre) as evaluation indicators for lesion segmentation. Among them, IoU measures the degree of overlap between the prediction area of the model and the real area, which can objectively evaluate the positioning accuracy of the model for the segmentation target area, considers the location and size of the segmentation target, and is versatile and intuitive. DSC is also a measure of the degree of overlap between the predicted area and the real area, and it is more sensitive to the segmentation results of small targets. HD is a metric that measures the farthest distance between two point sets, which is used to evaluate the distance between the segmentation result and the real result, which can reflect the difference between the boundary of the segmentation result and the real boundary, so it plays an important role in some scenarios where boundary accuracy needs to be concerned. In a segmentation model, Pre can be used to measure the predictive accuracy of the model for the target region. Params are a spatial measure of the complexity of a model. In this paper, there are differences in lesion shape, size and location in stroke segmentation, novel coronavirus pneumonia lesion segmentation and skin cancer lesion segmentation, and the clinical application will limit the computational resources but still need to maintain high accuracy, so the above evaluation indicators are selected in this paper. The values of IoU and DSC range from [0,1], and the larger the value, the more overlapping parts, that is, the closer the prediction result is to the true label. True Positives (TP), False Positives (FP) and False Negatives (FN). The formula is as follows:

8 $$\begin{aligned} IoU= & {} \frac{TP}{TP+FP+FN} \end{aligned}$$

9 $$\begin{aligned} DSC= & {} \frac{2TP}{2TP+FP+FN} \end{aligned}$$

Pre reflects the proportion of TP in all predicted positive samples. TP and FP are the same as above, the equation is as follows (10):

10 $$\begin{aligned} Pre=\frac{TP}{TP+FP} \end{aligned}$$

HD is a measure of the maximum mismatch between set A and set B. The formula is as follows(11,12,13):

11 $$\begin{aligned} HD(A,B)= & {} \max \{h(A,B),h(B,A)\} \end{aligned}$$

12 $$\begin{aligned} h(A,B)= & {} \mathop {\max }\limits _{a\in A} \{\mathop {\min }\limits _{b\in B} {\parallel a-b\parallel }\} \end{aligned}$$

13 $$\begin{aligned} h(B,A)= & {} \mathop {\max }\limits _{b\in B} \{\mathop {\min }\limits _{a\in A} {\parallel b-a\parallel }\} \end{aligned}$$

where $\parallel \cdot \parallel$ represents a norm of 2, *h*(*A*, *B*) represents the maximum distance from point a in set A to point b in set B closest to it. Similarly, *h*(*B*, *A*) represents the maximum distance from point b in set B to point a in set A closest to it.

### Experimental details

Stroke segmentation dataset, COVID-SemiSeg dataset and ISIC 2018 dataset were trained for 300, 300 and 200 epochs, respectively. The batch size of all datasets is 12, the optimizer is Adam, and the learning rate is 0.001. The experimental environment programming language is python3.7, the deep learning framework is PyTorch1.8, CUDA11.1. The main packages are Numpy1.21, Tensorboard2.5, SimpleITK2.0, etc. Hardware environment: The CPU is 12900KF, the GPU is a single Nvidia GeForce RTX3090, and the memory is 24G. The loss function is Binary Cross Entropy (BCE) loss, such as Eq. (14) as follows:

14 $$\begin{aligned} L_{BCE} = {-\frac{1}{ N }} \sum _{ i =1}^ N \left( {y_{i}}\cdot {\log ( p_{i} )} + (\textrm{1} - y_{i} ) \cdot \log (\textrm{1} - p_{i} ) \right) \end{aligned}$$

Where $N$ is the number of pixels, $y_{i}$ is the label of pixel $i$, and $p_{i}$ is the probability of being predicted as a positive class.

### Ablation study

In order to fully explore the impact of DCD and TA on model performance, we conducted ablation research on stroke segmentation dataset. In this experiment, Attention U-Net is used as the baseline network, and DCD and TA are added to the encoder-decoder to explore the performance of the model. The experimental results are shown in Table 1. “$\downarrow$” means the smaller the better, “$\uparrow$” means the bigger the better. Bold fonts indicate optimal results, Italic fonts indicate suboptimal results. The numbers in brackets represent the difference compared with the baseline network experiment results. “+” or “−” respectively represent the increase or decrease of the corresponding index. “$\surd$” indicates that DCD or TA is used in the encoding-decoding process, and “$\times$” indicates that DCD or TA is not used in the encoding-decoding process.

**Table 1:** DTA-UNet ablation experiment table.

	Encoder	Decoder	Params(M)$\downarrow$	IoU$\uparrow$	DSC$\uparrow$	HD$\downarrow$	Pre$\uparrow$
Baseline	$\times$	$\times$	**34.8786**	0.6964	0.7915	2.5378	0.8280
DCD	$\surd$	$\times$	35.3429	0.7218	0.8140	2.4280	0.8707
			(+ 0.4643)	(+ 0.0254)	(+ 0.0225)	(- 0.1098)	(+ 0.0427)
	$\times$	$\surd$	35.1743	0.7090	0.8003	2.4712	0.8671
			(+ 0.2975)	(+ 0.0126)	(+ 0.0088)	(- 0.0666)	(+ 0.0391)
	$\surd$	$\surd$	35.6387	*0.7285*	*0.8243*	*2.4241*	0.8780
			(+ 0.7601)	*(+0.0321)*	*(*+ 0.0328)	*(- 0.1137)*	(+ 0.0500)
	$\surd$	$\times$	34.8801	0.7201	0.8138	2.4296	0.8855
			(+ 0.0015)	(+ 0.0237)	(+ 0.0223)	(- 0.1082)	(+ 0.0575)
TA	$\times$	$\surd$	*34.8798*	0.7065	0.8037	2.5146	0.8656
			*(+ 0.0012)*	(+ 0.0101)	(+ 0.0122)	(- 0.0232)	(+ 0.0376)
	$\surd$	$\surd$	34.8813	0.7211	0.8140	2.4276	*0.8891*
			(+ 0.0027)	(+ 0.0247)	(+ 0.0225)	(- 0.1102)	*(+ 0.0611)*
DCD+TA	$\surd$	$\surd$	**35.6414**	**0.7353**	**0.8314**	**2.4214**	**0.8893**
(ours)			**(+ 0.7628)**	**(+ 0.0389)**	**(+ 0.0399)**	**(- 0.1164)**	**(+ 0.0613)**

Significant values are in bold and italics.

As shown in Table 1, compared with only using DCD in a single process, using DCD in the encoding-decoding process at the same time, although the number of Params is increased by a small amount, the four segmentation indicators of IoU, DSC, HD and Pre are significantly improved. When the TA module is used at the same time in the process of encoding and decoding, the number of Params hardly increases, and the improvement of various segmentation indexes is optimistic, among which Pre is the most obvious, reaching 0.8891. When the DCD and TA modules are used in the encoding-decoding process at the same time, the number of Params only increases by 0.7628M, and the four segmentation indicators are all optimal, which can more effectively improve the performance of Attention U-Net. The results of ablation experiments show that the combination of TA and AG is beneficial for the network to pay more attention to the lesion, and the introduction of DCD can promote the network to learn more complex features, which is more conducive to the accurate segmentation of lesions. Therefore, this study takes the approach of using both DCD and TA in the encoding-decoding process.

### Compare the experimental results

#### AIS experimental results

In order to evaluate the superiority of DTA-UNet and other methods, it was compared with eight state-of-art or classic methods. The eight methods are U-Net^1^, UNet++^4^, CE-Net^11^, Attention U-Net^12^, Inf-Net^24^ , SmaAt-UNet^26^, ELU-Net^27^ and CMUNeXt^28^. As shown in Table 2: Compared with the above eight methods, DTA-UNet shows the best results on the four segmentation indicators. The three indicators of IoU, DSC and Pre increased by 0.0386, 0.0431 and 0.0345 respectively, which shows that DTA-UNet is very effective for stroke region segmentation. HD decreased by 0.0928, which shows that DTA-UNet can get more accurate boundaries compared to other methods. At the same time, DTA-UNet has increased the number of Params by 4.5979M, which shows that it sacrifices the complexity of the model in exchange for the improvement of segmentation performance. Furthermore, although ELU-Net and SmaAt-UNet does not perform well in stroke segmentation, their lightweight design stems from the introduction of deep separable convolution, which requires minimal computational overhead.

**Table 2:** Performance comparison of different methods on the stroke segmentation dataset.

Methods	Params (M)$\downarrow$	IoU $\uparrow$	DSC $\uparrow$	HD $\downarrow$	Pre $\uparrow$
U-Net^1^	31.0435	0.6967	0.7883	2.5142	0.8548
UNet++^4^	9.1633	0.6945	0.7946	2.5074	0.8104
	(-21.8802)	(-0.0022)	(+0.0063)	(-0.0068)	(-0.0444)
CE-Net^11^	29.0031	0.6631	0.7694	2.6132	0.8168
	(-2.0404)	(-0.0336)	(-0.0189)	(+0.099)	(-0.038)
Attention U-Net^12^	34.8786	0.6964	0.7915	2.5378	0.8280
	(+3.8351)	(-0.0003)	(+0.0032)	(+0.0236)	(-0.0268)
SmaAt-UNet^26^	*4.1114*	0.6951	0.7986	2.5761	0.8220
	*(-26.9321)*	(-0.0016)	(+0.0103)	(+0.0619)	(-0.0328)
Inf-Net^24^	29.9922	*0.7041*	*0.8012*	2.5370	0.8516
	(-1.5013)	*(+0.0074)*	*(+0.0129)*	(+0.0228)	(-0.0032)
ELU-Net^27^	**0.8002**	0.6918	0.7909	2.6166	0.8239
	**(-30.2433)**	(-0.0049)	(+0.0026)	(+0.1026)	(-0.0309)
CMUNeXt^28^	31.4918	0.6896	0.7804	*2.5054*	*0.8586*
	(+0.4483)	(-0.0071)	(-0.0079)	*(-0.0088)*	*(+0.0038)*
**DTA-UNet(ours)**	**35.6414**	**0.7353**	**0.8314**	**2.4214**	**0.8893**
	**(+4.5979)**	**(+0.0386)**	**(+0.0431)**	**(-0.0928)**	**(+0.0345)**

Significant values are in bold and italics.

**Figure 3:**
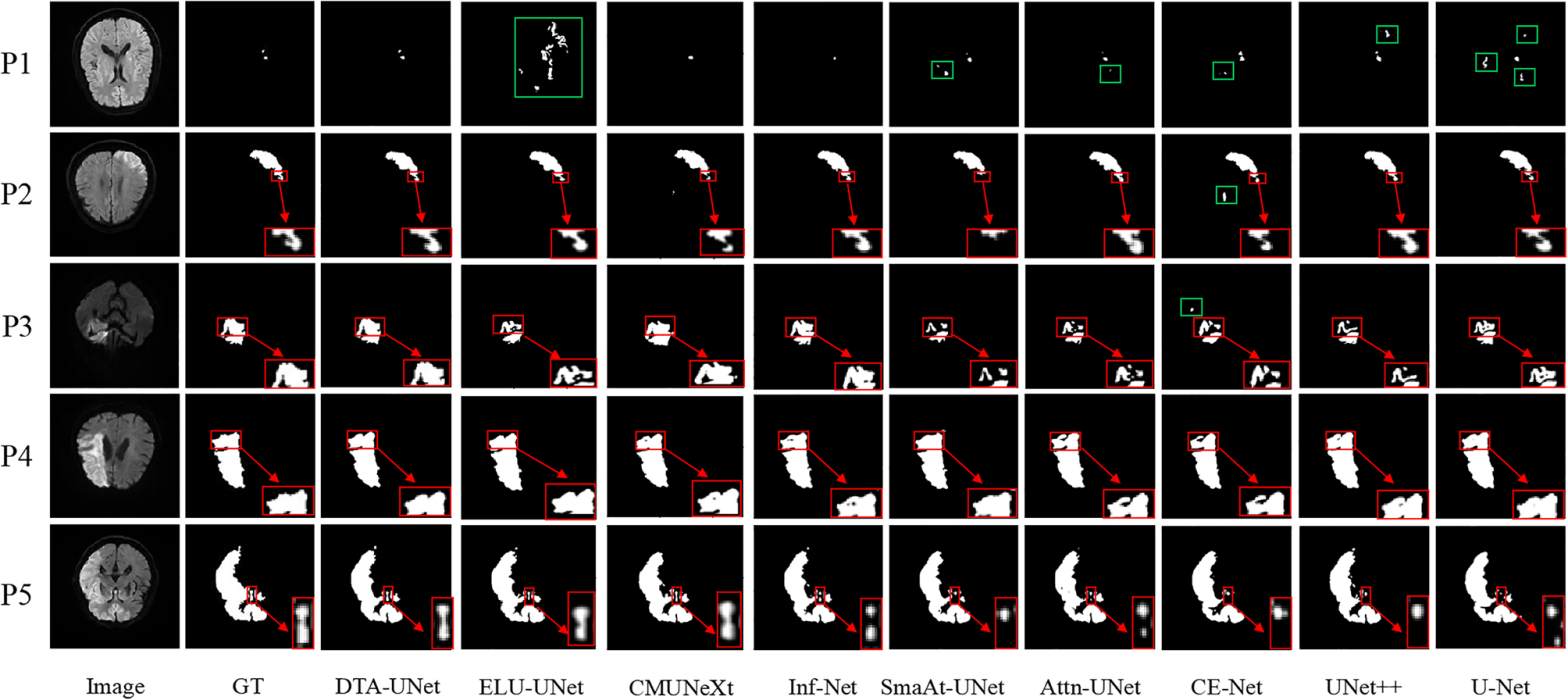
Visual comparison of stroke segmentation results. P* represents for patient*. The red box is the magnified image of the selected region, and the green box is the mis-segmentation.

The segmentation results on the stroke segmentation dataset are shown in Fig. 3, and each row corresponds to a different segmentation result of a selected patient. The first column is the original image, the second column is GT, The third column is the segmentation result of the DTA-UNet and the fourth to eleventh columns are the segmentation results of the above eight representative methods.

The segmentation results of 5 patients were randomly selected as shown in Fig. 3. For patient 1, DTA-UNet showed the best performance in small target segmentation, CMUNeXt and Inf-Net showed under-segmentation, and the other methods showed mis-segmentation. This may lead to missed or misdiagnosed clinically occurrence of the phenomenon. For medium and large lesions, such as patients 2–5, the segmentation results of DTA-UNet and Inf-Net are the closest to GT, and the other methods showed different degrees of under-segmentation or mis-egmentation. In addition, as shown in the red box, compared to other methods, DTA-UNet performs more prominently in details. This shows that the introduction of DCD and TA makes the model more focused on stroke regions and boundaries, thus strengthening the accurate segmentation of stroke.

#### COVID-semiseg segmentation experiment results

The segmentation results on the COVID-SemiSeg dataset are shown in Table 3. Compared with other methods, DTA-UNet and Inf-Net performed very well, and all indicators reached the optimal or suboptimal. This shows that the introduction of DCD and TA is also competitive in the segmentation of COVID-19 CT images. The DTA-UNet performed best on the two indicators of IoU and DSC, which increased by 0.0715 and 0.0672, respectively. HD took second place. HD dropped 0.3947, Pre improved 0.0593. It shows that DTA-UNet can well segment the COVID-19 infection regions, and Inf-Net, which integrates multiple edge attention modules, is friendly to the boundary extraction task.

**Table 3:** Performance comparison of different methods on the COVID-SemiSeg dataset.

Methods	IoU$\uparrow$	DSC$\uparrow$	HD$\downarrow$	Pre$\uparrow$
U-Net^1^	0.5329	0.676	7.2953	0.7128
UNet++^4^	0.5624	0.7111	7.1452	0.6917
	(+0.0295)	(+0.0351)	(-0.1501)	(-0.0211)
CE-Net^11^	0.5771	0.7198	7.1525	0.7435
	(+0.0442)	(+0.0438)	(-0.1428)	(+0.0307)
Attention U-Net^12^	0.5689	0.7137	7.1536	0.7516
	(+0.0360)	(+0.0377)	(-0.1417)	(+0.0388)
SmaAt-UNet^26^	0.5459	0.6913	7.1965	0.7421
	(+0.0130)	(+0.0153)	(-0.0988)	(+0.0293)
Inf-Net^24^	*0.5896*	*0.7329*	**6.8678**	*0.7816*
	*(+0.0567)*	*(+0.0569)*	**(-0.4275)**	*(+0.0688)*
ELU-Net^27^	0.5480	0.6938	7.1485	**0.7911**
	(+0.0151)	(+0.0178)	(-0.1468)	**(+0.0783)**
CMUNeXt^28^	0.5361	0.6832	7.4169	0.7621
	(+0.0032)	(+0.0072)	(+0.1216)	(+0.0493)
**DTA-UNet(ours)**	**0.6044**	**0.7432**	*6.9006*	**0.7721**
	**(+0.0715)**	**(+0.0672)**	*(-0.3947)*	**(+0.0593)**

Significant values are in bold and italics.

**Figure 4:**
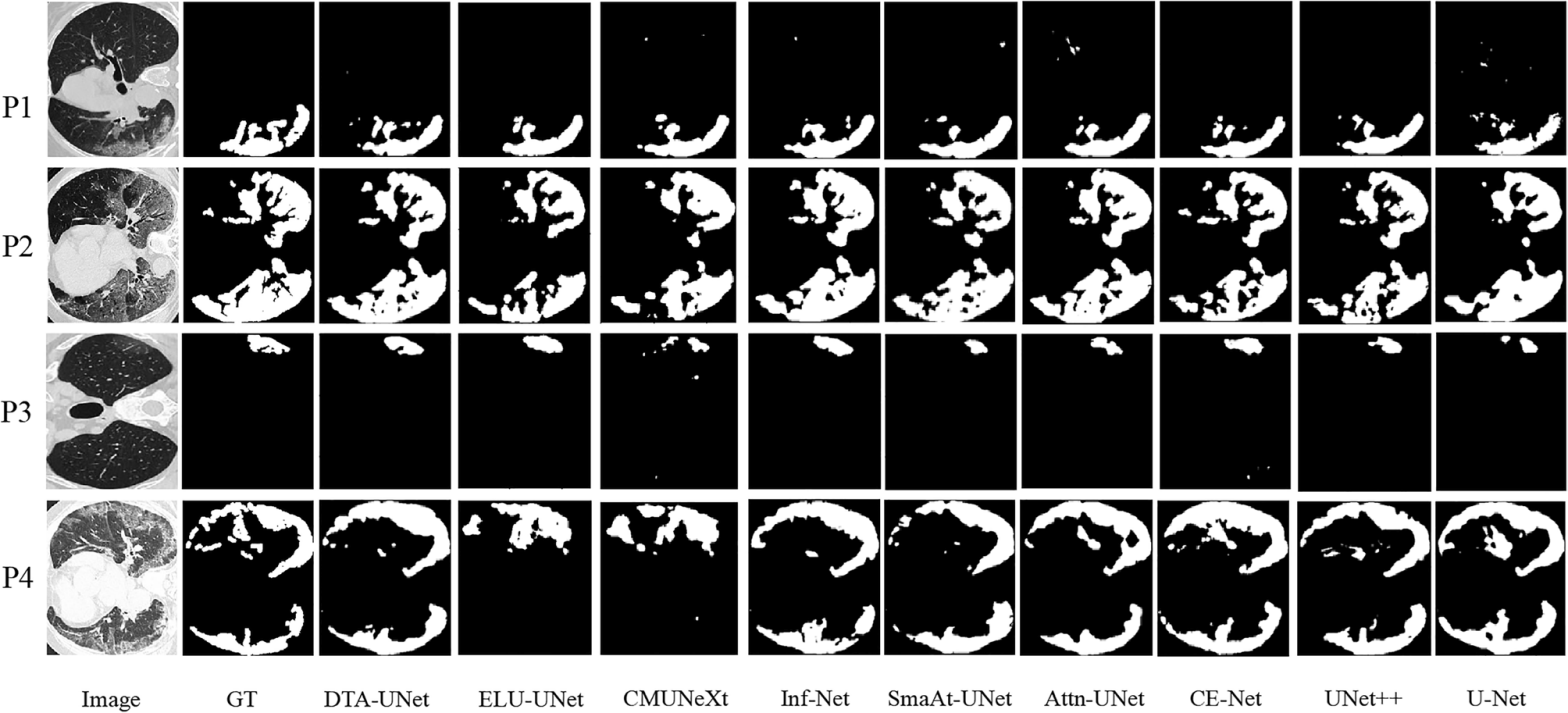
Visual comparison of segmentation results on the COVID-SemiSeg dataset.

The visualization results on the COVID-SemiSeg dataset are shown in Fig. 4. The segmentation results of DTA-UNet and Inf-Net are closer to GT, while other methods show limitations. In addition, as shown in patient 1, DTA-UNet has a slight advantage over Inf-Net for the segmentation of infected regions. This is attributed to the excellent feature extraction ability of DTA-UNet and higher attention to infected regions. As shown in patient 2, Inf-Net is slightly better at boundary segmentation, which further verifies the results in Table 3.

#### ISIC 2018 segmentation experiment results

The segmentation results on the ISIC 2018 dataset are shown in Table 4, DTA-UNet got the best results on the three indicators, and the Pre indicator also got second place. This further proves that the DTA-UNet model has better robustness and generalization ability.

**Table 4:** Performance comparison of different methods on the ISIC 2018 dataset.

Methods	IoU$\uparrow$	DSC$\uparrow$	HD$\downarrow$	Pre$\uparrow$
U-Net^1^	0.7932	0.8703	4.7411	0.8898
UNet++^4^	0.8007	0.8742	4.5411	0.8914
	(+0.0075)	(+0.0039)	(-0.2000)	(+0.0016)
CE-Net^11^	0.8029	0.8766	4.5317	0.8950
	(+0.0097)	(+0.0063)	(-0.2094)	(+0.0052)
Attention U-Net^12^	0.7964	0.8732	4.5686	0.9068
	(+0.0032)	(+0.0029)	(-0.1725)	(+0.0170)
SmaAt-UNet^26^	0.7797	0.8579	4.6570	0.8813
	(-0.0135)	(-0.0124)	(-0.0841)	(-0.0085)
Inf-Net^24^	*0.8109*	*0.8847*	*4.4216*	**0.9181**
	*(+0.0177)*	*(+0.0144)*	*(-0.3195)*	**(+0.0283)**
ELU-Net^27^	0.7672	0.8477	4.9227	0.8600
	(-0.026)	(-0.0226)	(+0.1816)	(-0.0298)
CMUNeXt^28^	0.7894	0.8677	4.7437	0.8791
	(-0.0038)	(-0.0026)	(+0.0026)	(-0.0107)
DTA-UNet(ours)	**0.8219**	**0.8864**	**4.4106**	*0.9085*
	**(+0.0197)**	**(+0.0161)**	**(-0.3305)**	*(+0.0187)*

Significant values are in bold and italics.

**Figure 5:**
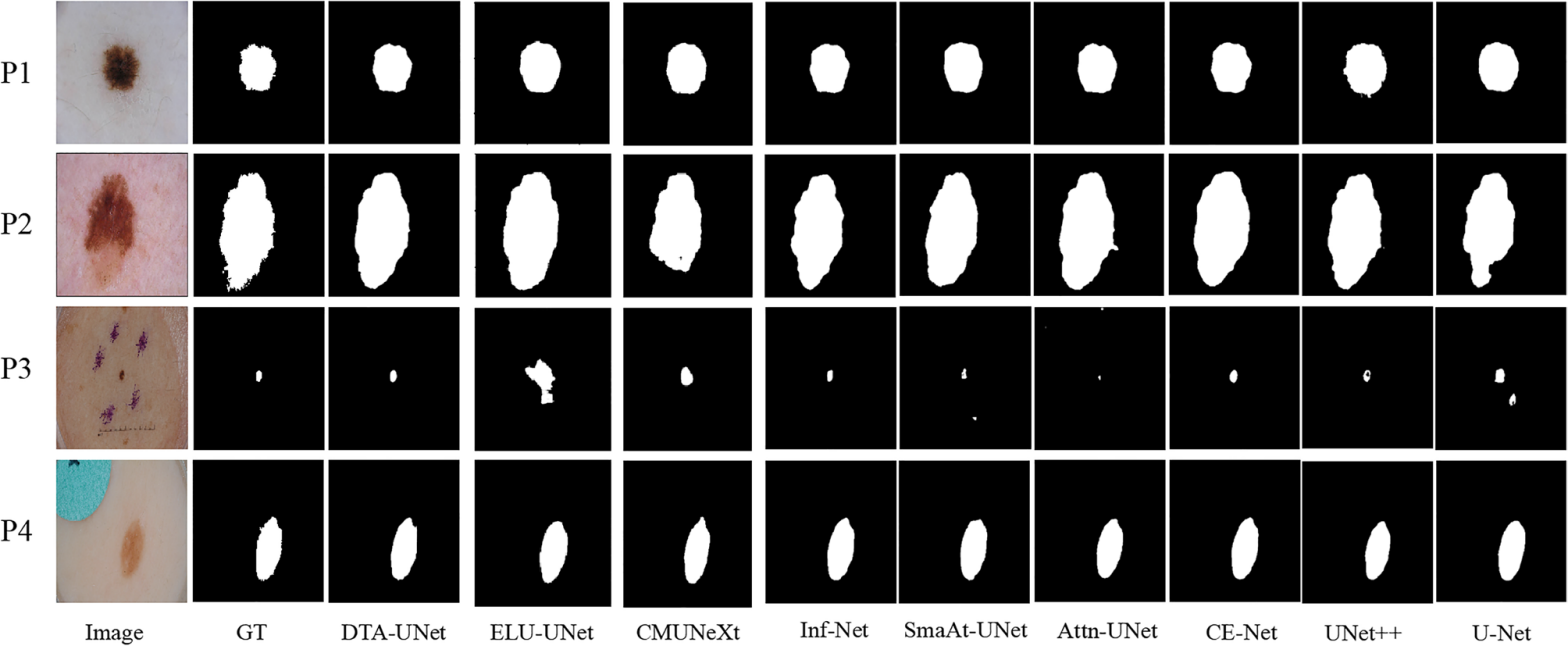
Visual comparison of segmentation results on the ISIC 2018 dataset.

The visual segmentation results on the ISIC 2018 dataset are shown in Fig. 5. For all dermoscopic images, DTA-UNet can effectively segment skin lesions, and combined with the segmentation index, the advantages of the DTA-UNet model are further verified. In contrast, such as patients 1, 2, and 4, Inf-Net is slightly inferior in the precise segmentation of boundaries. In addition, as shown in patient 3, although the baseline Attention U-Net recognized the lesion, it was under-segmented. This shows that DCD and TA are very necessary for the improvement of Attention U-Net.

In summary, DTA-UNet shows good performance on three different medical image segmentation datasets, reflecting good universality. This is attributed to the excellent feature extraction ability of DCD and the focus of TA on lesions in DTA-UNet.

## Conclusion

In this paper, we propose a universal deep learning model DTA-UNet for lesion segmentation. First, we replace the conventional convolution in Attention U-Net with DCD, which enhances the expressive ability of the model. Then, We use the combination of TA and AG to pay more attention to the target, effectively remove redundant information in the channel and space, and enhance the segmentation performance of the model. Compared with the current mainstream methods, DTA-UNet has shown excellent performance on three datasets of different image types and different disease types. However, it also adds some computational overhead, so how to make it lightweight will be explored in the future. In addition, transfer learning technology with the help of theoretical support can be used to extend the model to datasets with different distributions, reducing training costs. Therefore, combining the model proposed in this paper with transfer learning to solve new tasks is also an important research direction in the future.

## Data Availability

The data that support the findings of this study are available from the corresponding author, upon reasonable request.
